# Low serum Metrnl levels are associated with increased risk of sarcopenia in the older adults

**DOI:** 10.1007/s41999-024-01074-y

**Published:** 2024-10-03

**Authors:** Zhi-Yue Wang, Yi-Min Li, Jian-Jun Yan, Quan Wang, Can Zhao, Xiang Lu, Zheng-Kai Shen, Jin-Shui Xu, Wei Gao

**Affiliations:** 1 Department of Geriatrics, Zhongda Hospital, School of Medicine, Southeast University, Nanjing, 210009 China; 2 Jiangsu Province Center for Disease Control and Prevention, Nanjing, 210009 China; 3 Department of Cardiology, Sir Run Run Hospital, Nanjing Medical University, Nanjing, China; 4 Department of Cardiology, The People’s Hospital of Rugao, Rugao, China; 5 Department of Geriatrics, Sir Run Run Hospital, Nanjing Medical University, Nanjing, China; 6 Department of Geriatrics, Yancheng First Hospital, Affiliated Hospital of Nanjing University Medical School, Yancheng, China

**Keywords:** Older adults, Metrnl, Sarcopenia, Serum

## Abstract

**Purpose:**

Sarcopenia is a geriatric syndrome characterized by progressive loss of muscle mass and function. Meteorin-like (Metrnl) is a secretory protein that has protective effects on skeletal muscle injury. However, the association of Metrnl level with sarcopenia remains unclear.

**Methods:**

A total of 772 community-dwelling older adults (median age = 76 years), comprising 409 males and 363 females, from both urban and rural areas were enrolled. Serum Metrnl was measured by enzyme-linked immunosorbent assay. Appendicular skeletal muscle mass index (ASMI), grip strength, and gait speed were measured for the assessment of sarcopenia.

**Results:**

We found that serum Metrnl levels were lower in patients with sarcopenia [median (IQR) = 180.1 (151.3–220.3) pg/mL] than older adults without sarcopenia [211.9 (163.2–270.0) pg/mL, *P* < 0.001]. Receiver-operating characteristic curve analysis showed that the optimal cut-off value of serum Metrnl level that predicted sarcopenia was 197.2 pg/mL with a sensitivity of 59.2% and a specificity of 63.8% (AUC = 0.63, 95% CI = 0.59–0.67, *P* < 0.001). Multivariate logistic regression analyses showed that lower serum Metrnl level (< 197.2 pg/mL) was significantly associated with increased risk of sarcopenia (adjusted OR = 2.358, 2.36, 95% CI = 1.528–3.685, *P* < 0.001). Moreover, serum Metrnl concentration was positively correlated with the components of sarcopenia including ASMI (*r* = 0.135, *P* < 0.001), grip strength (*r* = 0.102, *P* = 0.005), and gait speed (*r* = 0.106, *P* = 0.003).

**Conclusions:**

Taken together, our findings demonstrate that low serum Metrnl level is correlated with increased risk of sarcopenia in the older adults.

**Supplementary Information:**

The online version contains supplementary material available at 10.1007/s41999-024-01074-y.

## Key summary points

**Aim:**

To investigate the association of serum Metrnl levels with the risk and severity of sarcopenia in a Chinese population of community-dwelling older adults.

**Findings:**

Our present study showed for the first time that serum Metrnl was positively correlated with the components of sarcopenia, including skeletal muscle mass, grip strength, and gait speed. Low serum Metrnl level (< 197.2 pg/mL) was associated with increased risk of sarcopenia in the older adults.

**Message:**

Addition of Metrnl to the traditional screening tools may help to facilitate the risk stratification for the patients with sarcopenia.

**Supplementary Information:**

The online version contains supplementary material available at 10.1007/s41999-024-01074-y.

## Introduction

Sarcopenia is an important geriatric syndrome characterized by loss of skeletal muscle mass accompanied by declined muscle strength and/or reduced physical performance [1]. The overall prevalence of sarcopenia is around 10% in the community-dwelling adults aged 60 years and older [2]. In China, the prevalence of sarcopenia ranges from 9.8% to 18.6% [3–5]. Sarcopenia can result in functional impairment and physical disability, leading to poor life quality and increased health care costs for the older adults [6]. The diagnosis of sarcopenia requires the measurements of muscle mass, muscle strength, and physical performance. However, due to the differences of age, sex, disease status, the degree of cooperation, cut-off values, etc*.*, the accuracy and sensitivity of the diagnosis remains unsatisfactory [7]. Although considerable efforts have been made to search the potential biomarkers for sarcopenia, the practicable non-invasive biomarkers for the early identification for sarcopenia are still limited.

Meteorin-like (Metrnl) has been identified as a myokine which can be induced upon exercise in skeletal muscle [8–10]. Metrnl treatment can alleviate high-fat-diet-induced inflammation and insulin resistance, and improve glucose metabolism in skeletal muscle [11, 12]. Metrnl is also increased and necessary for the muscle regenerative process in injured skeletal muscle through its anti-inflammatory effects [13]. Intriguingly, the skeletal expression of Metrnl was decreased with age, while intramuscular administration of recombinant Metrnl could promote the regeneration of muscle in aged mice [14]. These results suggest that Metrnl may act as a protective myokine in the pathogenesis of sarcopenia. However, whether the circulating level of Metrnl is correlated with the risk of sarcopenia remains unclear. Therefore, the present studies aimed to investigate the association of serum Metrnl levels with the risk and severity of sarcopenia in a Chinese population of community-dwelling older adults.

## Methods

### Study participants

A total of 772 older adults aged ≥ 65 years comprising of 499 patients with sarcopenia and 323 sex-matched subjects without sarcopenia were recruited from both rural and urban regions as previously described [15]. The rural participants were enrolled from Yuetang Medical Center in Yangzhou, Jiangsu Province, while the urban participants were from Maigaoqiao Community Medical Center in Nanjing, Jiangsu Province, respectively. Participants with the following conditions were excluded: (a) unable to move independently or failure to maintain in supine position; (b) unable to complete the specified actions due to nervous system diseases or bone and joint diseases or cardiopulmonary insufficiency; (c) severe renal insufficiency (creatinine clearance rate < 60 mL/min) or severe liver damage (transaminase increased more than 2 times); (d) malignant tumor. This study was performed in accordance with the principles outlined in the Declaration of Helsinki and approved by the Ethics Committee of Sir Run Run Hospital, Nanjing Medical University (Approval No. 2019-SR-S041). Written informed consent was obtained from each participant.

### Data collection

Venous blood sample was collected in the early morning after an overnight fasting and separated into serum and cellular fractions within 2 h. The serum was stored at − 80 °C before further analysis. Fasting blood glucose (FBG), alanine transaminase (ALT), aspartate aminotransferase (AST), total bilirubin (TBil), serum creatinine (SCr), blood urea nitrogen (BUN), total cholesterol (TC), triglyceride (TG), low-density lipoprotein-cholesterol (LDL-C), high-density lipoprotein-cholesterol (HDL-C), and hypersensitive C-reactive protein (hs-CRP) were measured. Participants who smoked more than 1 cigarette per day during the previous 12 months were classified as current smokers. Current drinkers were defined as those who drank alcohol at least once per day during the last 12 months [16].

### Assessment of sarcopenia

Sarcopenia was diagnosed according to the latest criteria of the Asian Working Group for Sarcopenia (AWGS) 2019 [1]. Muscle mass was measured by the method of bioelectrical impedance analysis (BIA) using Inbody S10 (Inbody, Korea). Appendicular skeletal muscle mass index (ASMI) was calculated as ASM divided by height squared in meters (ASM/height^2^). Low muscle mass was defined as an ASMI of less than 7.0 kg/m^2^ in men and 5.7 kg/m^2^ in women [1]. Grip strength was measured using a dynamometer (CAMRY EH101, China). Low handgrip strength was defined as < 28 kg in men and < 18 kg in women [1]. Usual gait speed on a 6-m course was used to test the physical performance. Slow walking speed was defined as a walking speed less than 1 m/s [1]. Patients with low muscle mass combined with low muscle strength or low physical performance were considered as moderate sarcopenia. Patients with low muscle mass combined with low muscle strength plus low physical performance were considered as severe sarcopenia [1].

### Serum metrnl measurements

Serum Metrnl levels were measured using an enzyme-linked immunosorbent assay (ELISA) kit (DY7867-05, R&D, USA) according to the manufacturer’s protocol. The intra-assay and inter-assay coefficients of variance were 2.55% and 3.42%, respectively. The analytic sensitivity of the assays was 15.625 pg/mL.

### Statistical analysis

The Kolmogorov–Smirnov test was used to test the normality of continuous variables, which were described as median (interquartile range, IQR). Mann–Whitney *U* test was used for determining the differences between two groups. Pearson *χ*^*2*^ test was used to compare qualitative variables represented as frequencies. Receiver-operating characteristic (ROC) curve analysis was used to determine the optimum cut-off value of serum Metrnl level best-predicting sarcopenia. Spearman correlation was used to calculate correlations between the clinical variables. Univariate analysis and multivariate logistic regression analysis were taken to determine the variables that contributed to the presence of sarcopenia. Odds ratios (ORs) and 95% confidence intervals (CIs) were calculated. All tests were two sided, and *P* < 0.05 was considered to be statistically significant. All analyses were performed using SPSS 28.0 (IBM, Chicago, IL).

## Results

### Characteristics of the study participants

This study population consisted of 772 older adults including 431 rural participants and 341 urban participants. The characteristics of the participants are shown in Table 1. There were 260 and 189 patients with sarcopenia in the rural and urban region, respectively (e-Table 1). As expected, patients with sarcopenia had lower levels of ASMI, grip strength, and gait speed than the older adults without sarcopenia (*P* < 0.001). Compared with the non-sarcopenia participants, sarcopenia patients were older and thinner (*P* < 0.05), and had lower levels of ALT, TBil, and TG (*P* < 0.01) but higher levels of FBG, BUN, HDL-C, and hs-CRP (*P* < 0.01). In addition, patients with sarcopenia had higher proportion of diabetes when compared to those non-sarcopenia participants (*P* = 0.004). No significant differences were observed between sarcopenia patients and non-sarcopenia participants with respect to sex, smoking, drinking, hypertension, AST, TC, and LDL-C. Importantly, serum Metrnl levels were lower in patients with sarcopenia [median (IQR) = 180.1 (151.3–220.3) pg/mL] than older adults without sarcopenia [211.9 (163.2–270.0) pg/mL] (Table 1). Specifically, we found that serum levels of Metrnl were lower in patients with sarcopenia both in rural [170.1 (133.1–215.3) pg/mL *vs.* 204.4 (159.6–255.2) pg/mL, *P* < 0.001] and urban [184.9 (156.1–220.0) pg/mL *vs.* 221.7 (171.4–308.1) pg/mL, *P* < 0.001] participants (e-Table 1).

**Table 1:** The characteristics of the enrolled subjects

Variables	Non-sarcopenia (*n* = 323)	Sarcopenia (*n* = 449)	*P*
Age, years	75.0 (71.0–80.0)	77.0 (70.0–82.0)	0.014
Male, *n* (%)	164 (50.8)	245 (54.6)	0.333
BMI, kg/m^2^	25.18 (23.18–27.17)	22.25 (19.90–23.89)	< 0.001
Smokers, *n* (%)	40 (12.4)	63 (14.0)	0.578
Drinkers, *n* (%)	32 (9.9)	49 (10.9)	0.741
Hypertension, *n* (%)	145 (44.9)	186 (41.4)	0.375
Diabetes, *n* (%)	38 (11.8)	89 (19.8)	0.004
FBG, mmol/L	5.50 (5.10–6.05)	5.62 (5.18–6.55)	0.008
ALT, U/L	16.24 (12.25–22.10)	14.70 (10.91–19.68)	0.001
AST, U/L	21.80 (19.0–27.0)	21.76 (18.02–26.94)	0.374
TBil, μmol/L	13.17 (10.70–16.65)	12.20 (9.20–15.71)	0.001
SCr, μmol/L	67.25 (53.22–81.94)	68.50 (52.65–84.3)	0.482
BUN, mmol/L	5.42 (4.53–6.27)	5.68 (4.62–6.98)	0.004
TC, mmol/L	4.78 (4.21–5.46)	4.91 (4.29–5.47)	0.350
TG, mmol/L	1.40 (1.04–1.90)	1.15 (0.88–1.66)	< 0.001
LDL-C, mmol/L	2.40 (1.86–2.87)	2.41 (1.87–3.00)	0.428
HDL-C, mmol/L	1.39 (1.22–1.58)	1.48 (1.24–1.74)	< 0.001
hs-CRP, mg/L	2.06 (1.68–4.03)	4.82 (1.93‐6.23)	< 0.001
Metrnl, pg/mL	211.9 (163.2–270.0)	180.1 (151.3–220.3)	< 0.001
Grip, kg	27.30 (22.20–33.10)	17.80 (15.40–24.30)	< 0.001
Males	33.10 (29.60–36.20)	23.80 (20.60–26.10)	< 0.001
Females	22.20 (19.70–24.70)	15.60 (13.33–16.70)	< 0.001
Gait speed, m/s	1.06 (0.98–1.16)	0.90 (0.79–1.04)	< 0.001
Males	1.00 (1.00–1.19)	0.91 (0.80–1.04)	< 0.001
Females	1.06 (0.98–1.13)	0.90 (0.74–1.01)	< 0.001
ASMI, kg/m^2^	7.08 (6.32–7.60)	5.64 (5.20–6.48)	< 0.001
Males	7.56 (7.14–7.99)	6.29 (5.91–6.71)	< 0.001
Females	6.34 (6.00–6.86)	5.19 (4.79–5.48)	< 0.001

*ALT* alanine transaminase, *ASMI* appendicular skeletal muscle mass index, *AST* aspartate aminotransferase, *BMI* body mass index, *BUN* blood urea nitrogen, *FBG* fasting blood glucose, *HDL-C *high-density lipoprotein cholesterol, *hs-CRP* hypersensitive C-reactive protein, *LDL-C* low-density lipoprotein cholesterol, *Metrnl* Meteorin-like, *Scr* serum creatinine, *TBil* total bilirubin, *TC* total cholesterol, *TG* triglyceride

### Association of serum metrnl with the risk of sarcopenia

**Fig. 1:**
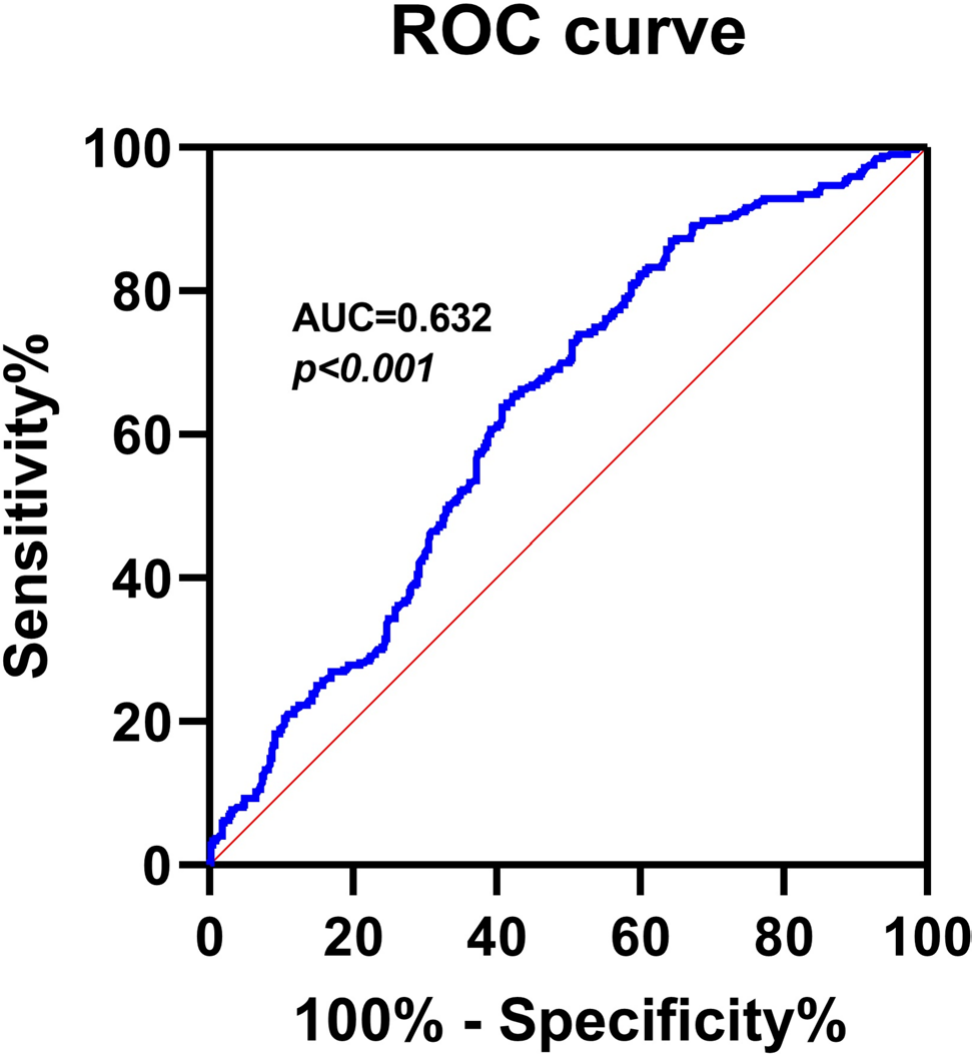
Receiver-operating characteristic (ROC) curves for the diagnostic accuracy of Serum Metrnl for sarcopenia (*n* = 772). AUC: Area Under Curve

To analyze the relationship between Metrnl and the risk of sarcopenia, we first investigated the correlation between Metrnl and clinical variables. As shown in Table 2, serum Metrnl level was negatively correlated with age, BMI, FBG, BUN, TG, HDL-C, and hs-CRP. ROC curve analysis indicated that the optimal cut-off value of serum Metrnl level that predicted sarcopenia was 197.2 pg/mL with a sensitivity of 59.2% and a specificity of 63.8% (AUC = 0.63, 95% CI = 0.59–0.67, *P* < 0.001) (Fig. 1). Univariate logistic regression analyses suggested a panel of variables that may associated with the risk of sarcopenia, including age, BMI, diabetes, FBG, TG, HLD-C, hs-CRP, and serum Metrnl level (e-Table 2). Further multivariate logistic regression analyses showed that low serum Metrnl level (< 197.2 pg/mL) was significantly associated with increased risk of sarcopenia (adjusted OR = 2.358, 95% CI = 1.528–3.685, *P* < 0.001) even after adjustment for the above potential confounding factors (Table 3). Similar results were observed when using serum Metrnl concentration as a continuous variable (adjusted OR = 1.006, 95% CI = 1.004–1.008, *P* < 0.001) (Table 3).

**Table 2:** Spearman’s correlation between serum Metrnl and clinical variables

Variables	Metrnl	
(*n* = 772)	*r*	*P*
Age	– 0.120	0.001
BMI	– 0.083	0.021
FBG	– 0.095	0.008
ALT	0.025	0.489
AST	– 0.036	0.315
TBil	0.068	0.060
Scr	0.060	0.097
BUN	– 0.124	0.001
TC	– 0.062	0.083
TG	– 0.090	0.013
LDL-C	– 0.002	0.950
HDL-C	– 0.109	0.002
hs-CRP	– 0.435	< 0.001

*ALT* alanine transaminase, *ASMI* appendicular skeletal muscle mass index, *AST* aspartate aminotransferase, *BMI* body mass index, *BUN* blood urea nitrogen, *FBG* fasting blood glucose, *HDL-C* high-density lipoprotein cholesterol, *hs-CRP* hypersensitive C-reactive protein, *LDL-*C low-density lipoprotein cholesterol, *Metrnl* Meteorin-like, *Scr* serum creatinine, *TBil* total bilirubin, *TC* total cholesterol, *TG* triglyceride

**Table 3:** Associations of serum Metrnl with the risk of sarcopenia

	Continuous		Categorical	
	OR (95% CI)	*P*	OR (95% CI)	*P*
Crude model	1.007 (1.005–1.009)	< 0.001	2.559 (1.906–3.437)	< 0.001
Adjusted model	1.006 (1.004–1.008)	< 0.001	2.358 (1.528–3.685)	< 0.001

The adjusted model included age, BMI, FBG, BUN, TG, HDL-C, and hs-CRP

*BMI* body mass index, *BUN* blood urea nitrogen, *CI* confidence interval, *FBG* fasting blood glucose, *HDL-C* high-density lipoprotein cholesterol, *hs-CRP* hypersensitive C-reactive protein, *Metrnl* Meteorin-like, *OR* odds ratio, *TG* triglyceride

### Stratification analyses for the association of serum metrnl with the risk of sarcopenia

Since previous studies reported that circulating Metrnl levels were correlated with diabetes and obesity [17, 18], stratified analyses were conducted according to age, sex, diabetes, and BMI. As shown in Table 4, the association of low Metrnl levels with increased risk of sarcopenia remained significance both in men (adjusted OR = 3.052, 95% CI = 1.794–4.482, *P* < 0.001) and women (adjusted OR = 1.906, 95% CI = 1.146–3.027, *P* = 0.015), as well as in the overweight (adjusted OR = 2.892, 95% CI = 1.638–4.965, *P* < 0.001) and normal weight (adjusted OR = 2.265, 95% CI = 1.552–3.683, *P* = 0.001) participants. By contrast, low serum Metrnl level was correlated with increased risk of sarcopenia only in the older adults younger than 80 years (adjusted OR = 3.248, 95% CI = 1.763–4.927, *P* < 0.001) and without diabetes (adjusted OR = 2.463, 95% CI = 1.672–3.774, *P* < 0.001).

**Table 4:** Stratification analyses for the association of serum Metrnl with the risk of sarcopenia

Variables	Continuous				Categorical			
	Crude OR (95% CI)	*P*	Adjusted OR (95% CI)	*P*	Crude OR (95% CI)	*P*	Adjusted OR (95% CI)	*P*
Age								
< 80 (*n* = 525)	1.008 (1.006–1.011)	< 0.001	1.008^a^ (1.005–1.013)	< 0.001	2.639 (1.850–3.765)	< 0.001	3.248^a^ (1.763–4.927)	< 0.001
≥ 80 (*n* = 247)	1.003 (0.999–1.007)	0.111	1.002^a^ (0.997–1.006)	0.417	2.228 (1.299–3.821)	0.004	1.835^a^ (0.972–2.785)	0.153
Sex								
Male (*n* = 363)	1.007 (1.004–1.011)	< 0.001	1.008^b^ (1.005–1.012)	< 0.001	2.744 (1.783–4.224)	< 0.001	3.052^b^ (1.794–5.482)	< 0.001
Female (*n* = 409)	1.007 (1.004–1.010)	< 0.001	1.007^b^ (1.002–1.009)	0.025	2.384 (1.590–3.574)	< 0.001	1.906^b^ (1.146–3.027)	0.015
Diabetes								
With (*n* = 127)	1.005 (0.999–1.010)	0.098	1.003^c^ (0.999–1.011)	0.205	1.344 (0.625–2.893)	0.449	1.455^c^ (0.596–3.568)	0.369
Without (*n* = 645)	1.008 (1.005–1.010)	< 0.001	1.006^c^ (1.003–1.008)	< 0.001	2.931 (2.123–4.046)	< 0.001	2.463^c^ (1.672–3.774)	< 0.001
BMI								
< 24.0 (*n* = 464)	1.005 (1.002–1.008)	0.001	1.005^d^ (1.001–1.007)	0.005	2.316 (1.506–3.562)	< 0.001	2.265^d^ (1.552–3.683)	< 0.001
≥ 24.0 (*n* = 308)	1.008 (1.004–1.012)	< 0.001	1.011^d^ (1.004–1.016)	< 0.001	2.634 (1.616–4.292)	< 0.001	2.892^d^ (1.638–4.965)	< 0.001

*BMI* body mass index, *BUN* blood urea nitrogen, *CI* confidence interval, *FBG* fasting blood glucose, *HDL-C* high-density lipoprotein cholesterol, *hs-CRP* hypersensitive C-reactive protein, *Metrnl* Meteorin-like, *OR* odds ratio, *TG* triglyceride

^a^The adjusted model included BMI, Diabetes, FBG, BUN, TG, HDL-C, and hs-CRP

^b^The adjusted model included age, BMI, Diabetes, FBG, BUN, TG, HDL-C, and hs-CRP

^c^The adjusted model included age, BMI, FBG, BUN, TG, HDL-C, and hs-CRP

^d^The adjusted model included age, Diabetes, FBG, BUN, TG, HDL-C, and hs-CRP

### Association of serum metrnl with the severity of sarcopenia

**Fig. 2:**
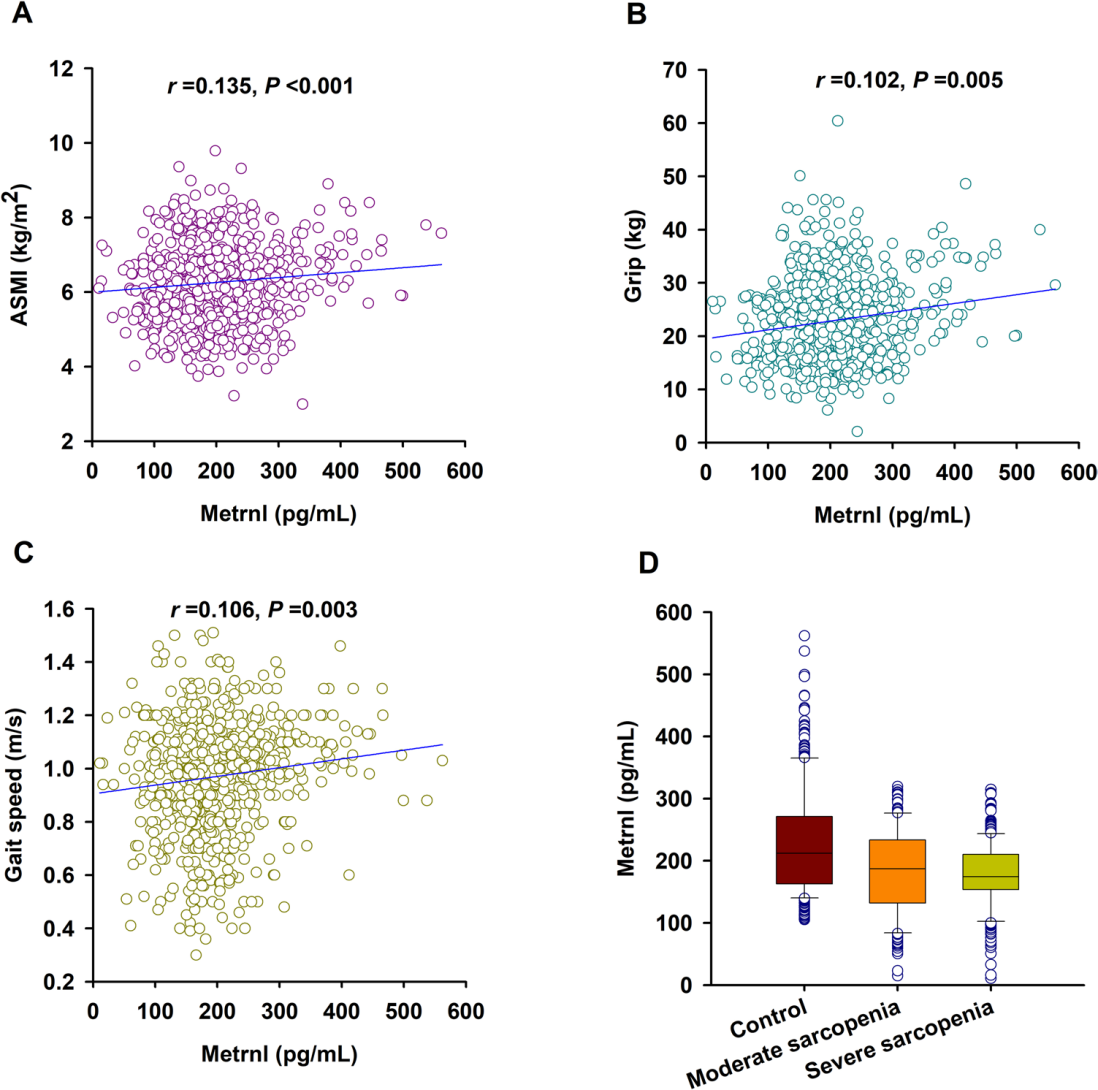
Association of serum Metrnl with the severity of sarcopenia. **A**–**C** Correlation of serum Metrnl level with ASMI (**A**), grip strength (**B**), and gait speed (**C**). **D** Serum Metrnl level in subjects without sarcopenia, patients with moderate sarcopenia, and patients with severe sarcopenia

Severe sarcopenia is characterized by loss of muscle mass combined with decreased muscle strength and low physical performance [1]. Although we found that serum Metrnl level was positively correlated with ASMI, grip strength, and gait speed (Fig. 2A–C); however, no significant difference of serum Metrnl level was observed between patients with moderate sarcopenia and those with severe sarcopenia [187.2 (132.1–233.0) pg/mL vs. 174.6 (153.6–210.2) pg/mL, *P* = 0.246] (Fig. 2D).

## Discussion

Our present study showed for the first time that serum Metrnl, a myokine with protective effects on skeletal muscle, was associated with sarcopenia in older adults. Among 772 community-dwelling older adults, serum Metrnl level was positively correlated with the components of sarcopenia, including skeletal muscle mass, grip strength, and gait speed. Low serum Metrnl level (< 197.2 pg/mL) was associated with increased risk of sarcopenia in the older adults.

Metrnl has been identified as a secreted protein which is homologous to the neutrophin Meteorin [19]. Previous clinical studies have linked decreased circulating levels of Metrnl levels with the increased susceptibility of diabetes [20, 21], metabolic dysfunction-associated fatty liver disease [22], coronary artery disease [23], heart failure [24], ischemic stroke [25], polycystic ovary syndrome [26], and inflammatory bowel disease [27]. Consistently, we here also showed that serum Metrnl levels were lower in patients with sarcopenia when compared to those non-sarcopenia participants. Moreover, low serum Metrnl levels were positively correlated with decreased muscle mass, grip strength, and gait speed, suggesting a predictive role of Metrnl concentration in muscle dysfunction. Indeed, despite a relatively low sensitivity and specificity, using < 197.2 pg/mL as cut-off value of serum Metrnl level could help to diagnose sarcopenia in the older adults. Our data demonstrate that low serum Metrnl level was associated with increased risk of sarcopenia in the older adults. Our results are consistent with recent animal studies showing that Metrnl could protect against aging-related sarcopenia in vivo [14, 28]. However, further prospective studies with larger sample sizes from different regions are still needed to confirm the predictive effect of circulating Metrnl level on the risk of sarcopenia.

Chronic inflammation has been identified as one of the key characteristics that associated with aging and aging-related diseases including sarcopenia [29]. The anti-inflammatory properties of Metrnl may contribute to the correlation between low Metrnl concentration and increased risk of sarcopenia [30]. As a myokine, Metrnl could activate M2 macrophage by inducing interleukin 4 (IL-4)/IL-13, which in turn suppresses the pro-inflammatory cytokines such as TNF‐α, IFN‐γ, and IL‐1β, and increases the expression of anti‐inflammatory genes IL‐10 and TGF‐β [8]. Similarly, another study showed that Metrnl could act as a critical regulator of muscle regeneration through promoting an anti-inflammatory/pro-regenerative environment via Signal transducer and activator of transcription 3 (Stat3)/insulin-like growth factor 1 (IGF-1) signaling [13]. Moreover, recombinant Metrnl treatment also alleviated lipid-induced inflammation in skeletal muscle cells through AMP-activated protein kinase (AMPK)- or peroxisome proliferator-activated receptor δ-dependent pathway [11]. Exercise, especially resistance exercise, has been proved as the most effective intervention for sarcopenia [31]. Intriguingly, Metrnl could be induced upon exercise in the skeletal muscle and mediate the protective effects of exercise on skeletal muscle function through inhibiting the activation of NLRP3 inflammasome [32, 33]. Consistently, we here also found that serum Metrnl was negatively correlated with the level of inflammatory marker hs-CRP (*r* = -0.435, *P* < 0.001). Considering that the expression of Metrnl in skeletal muscle decreases with age [14], the low Metrnl concentration might reflect an inflammatory status which is associated with the pathogenesis of sarcopenia in the older adults. Another potential underlying mechanism may relate to the beneficial effects of Metrnl on the glucose metabolism in skeletal muscle. Skeletal muscle is one of the major target organs for insulin signaling. Impaired insulin action not only causes insulin resistance but also promotes protein degradation and hampers protein synthesis in skeletal muscle cells, resulting in sarcopenia [34]. Previous study showed that Metrnl could stimulate the phosphorylation of AMPK, increase the glucose uptake, and improve glucose tolerance in skeletal muscle cells [11, 12]. Therefore, Metrnl might protect against the process of sarcopenia through attenuating insulin resistance. However, further in-depth investigation will be needed to confirm and elucidate the precise mechanism governing the protective effect of Metrnl on the development of sarcopenia.

Our stratified analyses showed that the correlation between low Metrnl levels and the risk of sarcopenia was prominent among participants without diabetes and younger than 80 years. Despite first identified as a peptide that has anti-diabetes ability in mice [8, 11, 12, 35, 36], the association of Metrnl concentration with diabetes in human remains controversial. Although a variety of studies found that circulating levels of Metrnl were lower and correlated to increased risk of diabetes [20, 21, 37]; however, other studies reported contradictory results showing that circulating Metrnl levels were higher in patients with diabetes [38–41]. In the present study, we did not find any significant difference in the serum level of Metrnl between patients with and without diabetes (median: 212.4 *vs.* 214.6 pg/mL, *P* = 0.238). Heterogeneity in the sample types, study population, and methodological design may cause the inconsistencies in these studies. In fact, diabetes per se may affect the relationship between Metrnl and sarcopenia, since insulin resistance is the common mechanism of diabetes and sarcopenia [42]. This may partially explain why the association of serum Metrnl with the risk of sarcopenia was only observed in participants without diabetes. Moreover, considering that the expression of Metrnl decreases with age [14], this change may minimize the causality of low Metrnl in the risk of sarcopenia in the adults older than 80 years. Nevertheless, the exact correlation between Metrnl level and the risk of sarcopenia in older adults with different statuses still needs to be further demonstrated.

Although first identified an association of low serum Metrnl level with increased risk of sarcopenia in the older adults, our present study should be interpreted within the context of some limitations. First, the participants in our study are only Chinese population, which cannot be representative of the global regional level. Second, although our study population was enrolled from rural and urban area, it was hard to exclude the possibility of selection and causality bias as a result of the cross-sectional nature. Thus, further prospective cohort studies will be needed to corroborate our findings. Third, we did not examine the correlation between serum Metrnl level and inflammatory factor to test the hypothesis that low Metrnl concentration may represent chronic inflammation status. Finally, although our results suggested that serum Metrnl might be served as a potential biomarker for sarcopenia, but the AUC was only 0.63 (< 0.7) with low sensitivity of 59.2% and specificity of 63.8%, indicating a relatively low predictive value.

## Conclusions

In summary, our findings demonstrate that low serum Metrnl level is correlated with the risk of sarcopenia in the older adults. Further studies of this biomarker may help to gain insight into the complex pathogenesis of sarcopenia.

## Supplementary information

Below is the link to the electronic supplementary material.

Supplementary file1 (DOCX 25 KB)

## Data Availability

The datasets used and/or analyzed during the current study are available at https://1drv.ms/x/c/2d38b0e521d2897a/EdgR4ak66MlDvfL4lK-SbNMBSXFjHsVujcDqPLRgltStqA?e=0U9Kxd.
