# Supplementary material for: Low serum Metrnl levels are associated with increased risk of sarcopenia in the older adults
Source: Eur Geriatr Med. 2024 Oct 3;15(6):1849–57. doi: 10.1007/s41999-024-01074-y (PMC11632026; doi:10.1007/s41999-024-01074-y)
Supplement: Supplementary file 1 — Supplementary file1 (DOCX 25 KB) [file 41999_2024_1074_MOESM1_ESM.docx]

**e-Table 1. The characteristics of the enrolled subjects in different regions.**

| **Variables** | **Urban (n=341)** | | |  | **Rural (n=431)** | | |
| --- | --- | --- | --- | --- | --- | --- | --- |
|  | **Non-Sarcopenia**  **(n=152)** | **Sarcopenia**  **(n=189)** | ***P*** |  | **Non-Sarcopenia**  **(n=171)** | **Sarcopenia**  **(n=260)** | ***P*** |
| **Age, years** | 78.0 (72.5-83.0) | 78.5 (70.0-83.0) | 0.319 |  | 73.0 (71.0-77.0) | 76.0 (71.0-81.0) | <0.001 |
| **Male, n (%)** | 80 (60.2) | 114 (54.8) | 0.390 |  | 84 (44.2) | 131 (54.4) | 0.036 |
| **BMI, kg/m^2^** | 24.80 (23.10- 26.25) | 22.75 (20.48 - 23.90) | <0.001 |  | 25.90 (23.20-27.60) | 21.75 (19.60-23.70) | <0.001 |
| **Smokers, n (%)** | 18 (13.5) | 25 (12.0) | 0.807 |  | 22 (11.6) | 38 (15.8) | 0.268 |
| **Drinkers, n (%)** | 11(8.3) | 9 (4.3) | 0.202 |  | 21 (11.1) | 40 (16.6) | 0.133 |
| **Hypertension, n (%)** | 90 (67.7) | 129 (62.0) | 0.344 |  | 55 (28.9) | 57 (23.7) | 0.257 |
| **Diabetes, n (%)** | 26 (19.5) | 69 (33.2) | 0.009 |  | 12 (6.3) | 20 (8.3) | 0.552 |
| **FBG, mmol/L** | 5.66 (5.08 - 6.35) | 5.91 (5.46 - 7.04) | <0.001 |  | 5.43 (5.14-5.80) | 5.33 (5.05-5.92) | 0.287 |
| **ALT, U/L** | 16.32 (13.07-22.72) | 16.58 (12.68 - 21.61) | 0.897 |  | 15.80 (11.95-22.00) | 12.50 (8.88-17.20) | <0.001 |
| **AST, U/L** | 21.03 (18.82-26.65) | 21.18 (17.44-25.38) | 0.447 |  | 23.00 (19.00-27.50) | 22.00 (19.00-29.00) | 0.791 |
| **TBil, μmol/L** | 13.59 (11.09-17.25) | 12.33 (9.52-15.21) | 0.005 |  | 12.70 (10.25-16.05) | 12.05 (8.50-16.05) | 0.038 |
| **SCr, μmol/L** | 78.17 (68.15-90.28) | 76.91 (64.45-94.98) | 0.646 |  | 55.70 (45.75-68.20) | 57.60 (45.70-73.95) | 0.305 |
| **BUN, mmol/L** | 5.66 (4.62-6.66) | 6.14 (4.95-7.65) | 0.010 |  | 5.08 (4.40-5.96) | 5.17 (4.34-6.40) | 0.227 |
| **TC, mmol/L** | 4.74 (4.11-5.58) | 4.95 (4.17-5.53) | 0.344 |  | 4.88 (4.24-5.36) | 4.83 (4.38-5.39) | 0.801 |
| **TG, mmol/L** | 1.37 (1.00-1.96) | 1.12 (0.88-1.67) | 0.002 |  | 1.40 (1.10-1.85) | 1.19 (0.89-1.63) | <0.001 |
| **LDL-C, mmol/L** | 2.69 (2.22-3.35) | 2.88 (2.21-3.42) | 0.228 |  | 2.19 (1.74-2.54) | 2.10 (1.66-2.47) | 0.237 |
| **HDL-C, mmol/L** | 1.36 (1.17-1.52) | 1.42 (1.21-1.69) | 0.006 |  | 1.47 (1.27-1.60) | 1.56 (1.28-1.77) | 0.002 |
| **hs-CRP, mg/L** | 2.03 (1.62-3.96) | 4.75 (1.32-8.22) | <0.001 |  | 2.24 (1.76-4.11) | 5.13 (1.41-7.97) | <0.001 |
| **Metrnl, pg/mL** | 221.7 (171.4-308.1) | 184.9 (156.1-220.0) | <0.001 |  | 204.4 (159.6-255.2) | 170.1 (133.1-215.3) | <0.001 |
| **Grip, kg** | 29.80 (22.40-35.00) | 17.80 (15.70-24.95) | <0.001 |  | 25.90 (22.20-31.10) | 17.75 (14.95-23.90) | <0.001 |
| **Males** | 33.80 (29.90-37.28) | 24.30 (21.38-26.50) | <0.001 |  | 32.45 (29.60-35.30) | 23.00 (19.60-25.40) | <0.001 |
| **Females** | 21.50 (17.90-24.95) | 15.70 (13.58-16.70) | <0.001 |  | 22.75 (20.48-24.63) | 15.25 (12.90-17.00) | <0.001 |
| **Gait speed, m/s** | 1.1 (0.9-1.2) | 0.9 (0.8-1.1) | <0.001 |  | 1.1 (1.0-1.1) | 0.9 (0.8-1.0) | <0.001 |
| **Males** | 1.1 (0.9-1.2) | 0.9 (0.7-1.1) | <0.001 |  | 1.1 (1.0-1.2) | 0.9 (0.9-1.0) | <0.001 |
| **Females** | 1.1 (0.9-1.2) | 1.0 (0.8-1.1) | <0.001 |  | 1.1 (1.0-1.1) | 0.8 (0.7-1.0) | <0.001 |
| **ASMI, kg/m^2^** | 7.00 (6.35-7.60) | 5.80 (5.30-6.50) | <0.001 |  | 7.10 (6.30-7.60) | 5.60 (5.10-6.30) | <0.001 |
| **Males** | 7.45 (7.10-7.90) | 6.50 (6.10-6.80) | <0.001 |  | 7.58 (7.34-8.04) | 6.18 (5.62-6.66) | <0.001 |
| **Females** | 6.30 (6.00-6.70) | 5.25 (5.00-5.50) | <0.001 |  | 6.46 (6.01-6.90) | 5.08 (4.58-5.39) | <0.001 |

BMI, body mass index; FBG, fasting blood glucose; ALT, alanine transaminase; AST, aspartate aminotransferase; TBil, total bilirubin; SCr, serum creatinine; eGFR, estimated glomerular filtration rate; BUN, blood urea nitrogen; TC, total cholesterol; TG, triglyceride; LDL-C, low-density lipoprotein cholesterol; HDL-C, high-density lipoprotein cholesterol; hs-CRP, hypersensitive C-reactive protein; Metrnl, Meteorin-like; ASMI, appendicular skeletal muscle mass index.

**e-Table 2. Univariate analysis for the associations of clinical variables with the risk of sarcopenia.**

| **Variables** | **OR (95% CI)** | ***P*** |
| --- | --- | --- |
| **Age** | 1.025 (1.004-1.047) | 0.021 |
| **Male** | 1.164 (0.874-1.551) | 0.298 |
| **BMI** | 0.685 (0.643-0.730) | <0.001 |
| **Smokers**  **Drinkers**  **Hypertension**  **Diabetes** | 1.155 (0.755-1.766)  1.114 (0.696-1.783)  0.868 (0.650-1.159)  1.854 (1.230-2.795) | 0.507  0.653  0.337  0.003 |
| **FBG** | 1.174 (1.065-1.294) | 0.001 |
| **ALT** | 0.990 (0.978-1.003) | 0.122 |
| **AST** | 1.005 (0.991-1.018) | 0.484 |
| **TBil** | 0.978 (0.954-1.003) | 0.086 |
| **SCr** | 1.006 (1.000-1.011) | 0.068 |
| **BUN** | 1.146 (1.054-1.246) | 0.001 |
| **TC** | 1.104 (0.964-1.265) | 0.154 |
| **TG** | 0.675 (0.556-0.820) | <0.001 |
| **LDL-C** | 1.093 (0.914-1.308) | 0.329 |
| **HDL-C** | 2.560 (1.646-3.981) | <0.001 |
| **hs-CRP** | 1.257 (1.013-1.418) | 0.002 |
| **Metrnl** | 1.007 (1.005-1.009) | <0.001 |

ALT, alanine transaminase; ASMI, appendicular skeletal muscle mass index; AST, aspartate aminotransferase; BMI, body mass index; BUN, blood urea nitrogen; CI, confidence interval; FBG, fasting blood glucose; HDL-C, high-density lipoprotein cholesterol; hs-CRP, hypersensitive C-reactive protein; LDL-C, low-density lipoprotein cholesterol; Metrnl, Meteorin-like; OR, odds ratio; Scr, serum creatinine; TBil, total bilirubin; TC, total cholesterol; TG, triglyceride.
